# PDK-1/AKT pathway as a novel therapeutic target in rhabdomyosarcoma cells using OSU-03012 compound

**DOI:** 10.1038/sj.bjc.6603952

**Published:** 2007-09-11

**Authors:** L Cen, F-C Hsieh, H-J Lin, C-S Chen, S J Qualman, J Lin

**Affiliations:** 1 Center for Childhood Cancer, Columbus Children's Research Institute, Department of Pediatrics, The Ohio State University, Columbus, OH 43205, USA; 2 Ohio State Biochemistry Program, The Ohio State University, Columbus, OH 43205, USA; 3 Division of Medical Technology, School of Allied Medical Professions, The Ohio State University, Columbus, OH 43205, USA; 4 Ohio State University Comprehensive Cancer Center, The Ohio State University, Columbus, OH 43205, USA; 5 Division of Medicinal Chemistry and Pharmacognosy, College of Pharmacy, The Ohio State University, Columbus, OH 43205, USA

**Keywords:** AKT, PDK-1, rhabdomyosarcoma, small molecular inhibitor, tissue microarray

## Abstract

Rhabdomyosarcoma (RMS) is the most common paediatric soft-tissue sarcoma including two major subtypes, alveolar rhabdomyosarcoma (ARMS) and embryonal rhabdomyosarcoma (ERMS). Increasing evidence suggests that oncogenesis of RMS involves multistages of signalling protein dysregulation which may include prolonged activation of serine/threonine kinases such as phosphoinositide-dependant kinase-1 (PDK-1) and AKT. To date, whether PDK-1/AKT pathway is activated in RMS is unknown. This study was to examine phosphorylation status of AKT and to evaluate a novel small molecular inhibitor, OSU-03012 targeting PDK-1 in RMS. We examined phosphorylation levels of AKT using ARMS and ERMS tissue microarray and immunohistochemistry staining. Our results showed phospho-AKT^Thr308^ level is elevated 42 and 35% in ARMS and ERMS, respectively. Phospho-AKT^Ser473^ level is also increased 43% in ARMS and 55% in ERMS. Furthermore, we showed that OSU-03012 inhibits cell viability and induces apoptosis in ARMS and ERMS cell lines (RH30, SMS-CTR), which express elevated phospho-AKT levels. Normal cells are much less sensitive to OSU-03012 and in which no detectable apoptosis was observed. This study showed, for the first time, that PDK-1/AKT pathway is activated in RMS and may play an important role in survival of RMS. PDK-1/AKT pathway may be an attractive therapeutic target for cancer intervention in RMS using OSU-03012.

Rhabdomyosarcoma (RMS) is the most common soft-tissue sarcoma of childhood. Approximately 350 new cases occur each year in the United States and they account for 4% of all childhood malignancies (Ries *et al*, 1999). Most of these tumours arise in the head and neck region, genitourinary tract and extremity. On the basis of histological criteria, it can be classified into two major subtypes, alveolar rhabdomyosarcoma (ARMS) and embryonal rhabdomyosarcoma (ERMS). Emerging evidence has been shown that the malignant growth of RMS involves a multistep process of signalling protein dysregulation that includes prolonged activation of serine/threonine kinases which may include 3-phosphoinositide-dependant kinase-1 (PDK-1)/AKT pathway. PDK-1 is a serine/threonine kinase that is activated in response to insulin and growth factor treatment by a mechanism involving phosphoinositide-3 kinase (PI3-K) (Mora *et al*, 2004). PDK-1 was identified by its ability to phosphorylate and activate AKT (Alessi *et al*, 1997; Stokoe *et al*, 1997), p70 S6 kinase (Alessi *et al*, 1998), and possibly protein kinase C (PKC) isozymes (Le Good *et al*, 1998; Balendran *et al*, 2000). PDK-1 can transform normal human cells and may be involved in invasion and metastasis process (Zeng *et al*, 2002; Xie *et al*, 2003, 2006). PDK-1 and its downstream target AKT are frequently phosphorylated and activated in multiple types of cancers (Cheng *et al*, 1992; Sun *et al*, 2001; Lin *et al*, 2005). Constitutive activation of PI3-K/PDK-1/AKT signalling mediates the survival signals and confers resistance to apoptosis induced by anticancer cytotoxic agents in human cancer cells (Page *et al*, 2000; Nesterov *et al*, 2001; Clark *et al*, 2002). Further, inhibition of PDK-1 using antisense oligonucleotides decreased cell proliferation and increased apoptosis in cancer cells which expresses constitutively active PDK-1/AKT pathway (Flynn *et al*, 2000). The effect of PDK-1 inhibition on cell proliferation and survival by antisense oligonucleotides implicates PDK-1 as a potential therapeutic target for human cancers including RMS. To date, the activation of PDK-1/AKT pathway in RMS has not been reported. Our results reported here demonstrates AKT was frequently phosphorylated and activated in ARMS and ERMS tissue microarray (TMA) indicating PDK-1/AKT pathway is activated in human RMS. Since there has been no significant improvement in the outcome for the treatment of RMS in the past 20 years, novel therapeutic methods are urgently needed. Given that PDK-1/AKT pathway is constitutively activated in RMS and may contribute to progression of the disease and possibly the anticancer drug resistance, there is a critical need to develop inhibitors of PDK-1/AKT pathway as potential treatment for RMS. We have recently developed a novel small molecule compound, OSU-03012, which targets PDK-1 pathway (Zhu *et al*, 2004). Therefore, we explored the potency of this compound for treatment of RMS. The compound was tested in RMS cell lines that express high levels of phospho-AKT, namely RH30 and SMS-CTR. Our results demonstrated OSU-03012 inhibited AKT phosphorylation. In addition, OSU-03012 led to cell viability decline and induced apoptosis in RMS cells (RH30 and SMS-CTR) but had minimal effect in normal human cells. These data showed the activation of PDK-1/AKT pathway in RMS and this pathway may play an important role in survival of RMS. These results suggest that PDK-1, which is an upstream regulator of AKT, may be an attractive therapeutic target for cancer intervention in RMS and OSU-03012 might be a potential therapeutic treatment for RMS patients, especially those cases with PDK-1/AKT pathway activated.

## MATERIALS AND METHODS

### Cell lines and cell culture

Human ARMS cell lines, RH3, RH30, CW9019 and ERMS cell lines, RD2 and SMS-CTR were maintained in DMEM supplemented with 10% fetal bovine serum (FBS; Fisher Scientific, Chicago, IL, USA).

### Tissue microarray slides and immunohistochemistry

Human RMS TMA slides were obtained from the Children's Oncology Group (COG). The ERMS TMA block contains 32 unique ERMS cases with two types of control tissues; one control tissue is normal skeletal muscle (five cases) and the other is ARMS (five cases). Each case averaged three tissue cores (range 1–9 cores), which were each 1.0 mm in diameter and randomly distributed throughout the block. The ARMS TMA also contains 32 unique cases with identical block design as defined above. In addition to five normal skeletal muscle control cases, five ERMS cases were included as controls. Rhabdomyosarcoma diagnoses were made via review by COG and STSC (Soft Tissue Sarcoma Committee) designated reviewers using the International Classification of Rhabdomyosarcomas (Qualman *et al*, 1998).

These slides were baked at 60°C for 1 h, deparaffinised in xylene three times, transferred through two changes of 100% ethanol, and then dehydrated with graded ethanol. Antigen retrieval was carried out by boiling the slides in a beaker filled with 10 mM sodium citrate (pH 6.0) or 1 mM EDTA (pH 8.0). Endogenous peroxidase activity was quenched by 10-min incubation in 3% hydrogen peroxide. After antigen retrieval, the slides were briefly rinsed with 0.1% Tween/1 × TBS (0.1% TBST) two times and then washed three times for 10 min each at room temperature to remove nonspecific background binding. The protein blocking were performed by incubating the specimens in 5% normal goat serum or normal horse serum in 0.1% TBST for 1 h. Primary antibody was applied at 4°C overnight (1 : 50 dilution of rabbit monoclonal anti-human phospho-specific antibodies from Cell Signaling Technology, Beverly, MA, USA and Santa Cruz Biotechnology Inc., Santa Cruz, CA, USA) in 0.1% TBST with normal serum. The phospho-specific antibodies we tested are phospho-AKT (Thr308), phospho-AKT (Ser473) (Cell Signaling Technology). After a series of TBS rinses as described above, bound antibody was subsequently detected using a VECTASTAIN ABC KIT from Vector Laboratories Inc. (Burlingame, CA, USA). The sections were then visualised with a 5–30 min incubation of 3-amino-9-ethylcarbazole (AEC) high-sensitivity substrate chromogen from DakoCytomation (Carpinteria, CA, USA). Finally, the slides were counterstained with haematoxylin and mounted with CRYSTAL/MOUNT™ (Biomeda Corp., Foster City, CA, USA) for long-term preservation.

### Evaluation of immunohistochemical staining

Immunohistochemical staining was scored under the microscope by eye. The staining intensity was graded relatively based on the following scale: 0, no staining; 1, weak staining; 2, moderate staining and 3, intense staining (Dolled-Filhart *et al*, 2003). For specimens that were uninterpretable or were missing in most of the cancer tissues, a score of not applicable (NA) was given. Scoring of the TMA was completed by at least two independent observers (LC and FH). Discrepant scores between the two observers were rescored to arrive at a single final score. In order for a tumour specimen to be considered positive, it had to have a score of 2 or greater from both observers.

### Cell viability assay

Cell viability was analysed by the [3-(4,5-dimethyl-2-thiazolyl)-2,5-diphenyl-2*H*-tetrazolium bromide] (MTT) assay in three replicates. Cells were grown in 10% FBS-supplemented DMEM in 96-well flat-bottomed plates overnight and were exposed to various concentrations of small molecule inhibitors dissolved in dimethylsulphoxide in the same medium. At the time of assay end point, MTT was added to the cultures and incubated for 3–4 h. Solubilisation buffer was then added. Plates were kept overnight in the dark to allow dissolving the formazan. Absorbance at 595 nm was determined on a plate reader. IC_50_, the drug concentration at which 50% growth inhibition is achieved, was calculated with WinNonlin Software (Scientific Consulting Inc., Apex, NC, USA).

### Western blot analysis

Total protein lysates (50–100 *μ*g per lane), as determined by BCA protein assay kit, were separated by 10% SDS–polyacrylamide gels and transferred onto PVDF membrane. Membranes were blotted with a 1 : 1000 dilution of antibodies against phospho-AKT (Ser473 or Thr308), total AKT, cleaved poly(ADP-ribose) polymerase (PARP) (Cell Signaling Technology), or phospho-p70 S6 kinase (Thr229) (R&D Systems Inc., Minneapolis, MN, USA). The same membranes were analysed with a 1 : 1000 dilution of anti-glyceraldehyde-3-phosphate dehydrogenase (GAPDH) monoclonal antibody (Chemicon International Inc., Temecula, CA, USA) as a protein loading control. All blots were incubated with 1 : 600 dilution of secondary fluorescein-linked anti-mouse or anti-rabbit antibody followed by incubation with 1 : 2500 dilution of alkaline phosphatase-conjugated anti-fluorescein antibody (Amersham Biosciences, Piscataway, NJ, USA). Blots were scanned with ImageQuant software using an ECF Western blotting detection system (Amersham Biosciences) on a Molecular Dynamics Storm PhosphorImager (Sunnyvale, CA, USA).

### Immunofluorescent staining

Cells were seeded on sterile coverslips in a six-well plate overnight. Cells were treated with 10 *μ*M OSU-03012 for 24 h and then were fixed with ice-cold methanol. Three washes followed fixation using 1 × PBS. During the third wash, coverslips were transferred onto a new six-well plate. Cells were blocked in 1 × PBS with 10% normal horse serum for 1 h and incubated with primary rabbit antibody that recognises cleaved-caspase-3 (Asp175) (Cell Signaling Technology) with 1 : 100 dilution overnight. Excess antibody was removed followed by three washes using 1 × PBS with constant agitation. Secondary goat anti-rabbit lgG (H+L) Alexa Fluo^R^ 594 antibody (Invitrogen, Carlsbad, CA, USA) with 1 : 1000 dilutions was incubated with 1% bovine serum albumin in 1 × PBS for 1 h. After three washes using 1 × PBS, nuclei were stained using 4′,6-diamidino-2-phenylindole dihydrochloride (100 ng ml^−1^) for 5 min, and rinsed three times using 1 × PBS. The fluorescence microscopic photographs were documented using LEICADM-IRB inverted fluorescent microscope (Leica Microsystems Inc., Bannockburn, IL, USA) with an attached Diagnostic RT-SE6 monochrome digital camera (Diagnostic Instruments Inc., Sterling Heights, MI, USA).

## RESULTS

### Increased frequency of phospho-AKT levels in RMS tissues and cells

**Figure 1:**
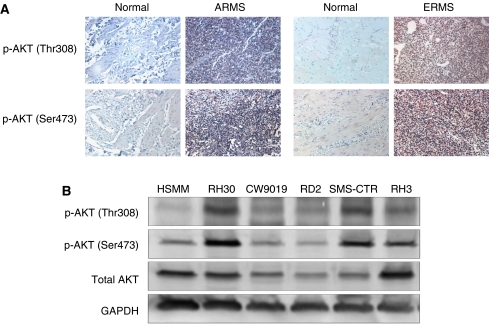
Expression of phosphorylated AKT in rhabdomyosarcoma (RMS) patient tissues and cell lines. (**A**) Immunohistochemical (IHC) staining of RMS tissue microarrays (TMAs) that contain both normal and cancer tissues using phospho-AKT (Thr308) and phospho-AKT (Ser473) antibodies. (**B**) The levels of phosphorylated AKT were examined using antibodies to threonine 308 and serine 473 in a series of RMS cell lines.

To determine whether PDK-1/AKT pathway is activated in human RMS, immunohistochemistry was used to detect the expression levels of phospho-AKT in ARMS and ERMS. Clinicopathological features of RMS patients are given in Table 1. The age of the patients in these cases are between 0 and 19 with mean 9.1 in ARMS and 5.8 in ERMS, and most of the cases are in stage III (Table 1). In each type of ARMS and ERMS, there are 32 cases along with 5 normal tissues as negative controls. Our results showed that phospho-AKT (Thr308) was moderate-to-strong expression in 42% of ARMS and 35% of ERMS cases (Figure 1A; Table 2). Phospho-AKT (Ser473) was moderate-to-strong expression in 43% of ARMS and 55% of ERMS cases (Figure 1A, Table 2). In contrast, normal tissues are mostly negative to weak staining. We also examined the relationship of phospho-AKT with clinicopathological parameters, such as tumour subtype, stage and age. But no significant correlation was found (data not shown). Since Thr308 of AKT is phosphorylated by PDK-1 (Alessi *et al*, 1997), these results suggest that PDK-1 is also activated in RMS. We also examined a couple of RMS cell lines for AKT phosphorylation. The cell lines include human skeletal muscle myoblasts (HSMM), ARMS cell line RH30, CW9019, RH3 and ERMS cell line RD2, SMS-CTR. The RH30 and SMS-CTR cells expressed the highest level of phospho-AKT (Thr308 and Ser473) respectively among alveolar and embryonal cell lines (Figure 1B). Therefore, they were further used to examine the inhibitory effects of OSU-03012 on RMS cells.

### Novel small molecule compound, OSU-03012 targeting PDK-1 inhibits AKT phosphorylation in RMS cells

**Figure 2:**
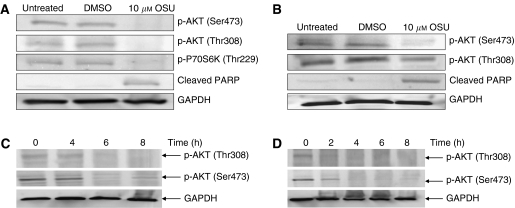
Effects of phosphoinositide-dependant kinase-1 (PDK-1) inhibitor OSU-03012 (OSU) on rhabdomyosarcoma (RMS) cell lines. (**A**) RH30 cells were treated for 8 h with 10 *μ*M OSU-03012. OSU-03012 downregulates AKT phosphorylation at both threonine 308 and serine 473. Phosphorylation of p70S6K at threonine 229, one of the downstream targets of PDK-1, was also inhibited by OSU-03012. Poly(ADP-ribose) polymerase (PARP) cleavage was measured with anti-cleaved-PARP antibody by western blotting. (**B**) SMS-CTR cells were treated as above. OSU-03012 inhibits AKT phosphorylation at both threonine 308 and serine 473. Poly(ADP-ribose) polymerase (PARP) cleavage was measured with anti-cleaved-PARP antibody by western blotting. (**C**) RH30 cells were treated with 10 *μ*M OSU-03012 for 4, 6 and 8 h respectively. AKT phosphorylation was inhibited in a time-dependent manner. (**D**) SMS-CTR cells were treated with 10 *μ*M OSU-03012 for 2, 4, 6 and 8 h respectively. Time course inhibition of phosphorylated-AKT was shown by western blot.

To study the inhibitory effects of small molecule inhibitor OSU-03012, that targets PDK-1, RMS cell line RH30 and SMS-CTR, which express elevated AKT phosphorylation levels at Thr308 (a PDK-1 phosphorylation site), were treated with 10 *μ*M OSU-03012 for 8 h, respectively. Our data showed that OSU-03012 treatment resulted in inhibition of AKT phosphorylation in both RH30 (Figure 2A) and SMS-CTR cell lines (Figure 2B) but did not inhibit ERK phosphorylation, which is independent of AKT pathway (data not shown). Phosphorylation of p70S6K at threonine 229, one of the downstream targets of PDK-1, was also inhibited by OSU-03012 in RH30 cells (Figure 2A), but much less inhibited in SMS-CTR cells (data not shown). To further study the kinetics of the inhibition caused by OSU-03012, RH30 and SMS-CTR cells were treated with 10 *μ*M OSU-03012 for different time course. Western blotting analysis showed that inhibition of p-AKT (Thr308) was observed as early as 2-h treatment in SMS-CTR cells (Figure 2D), whereas that was only seen at 6-h and 8-h treatment in RH30 cells (Figure 2C). In SMS-CTR, level of p-AKT (Ser473) slightly decreased after 2-h treatment and further inhibition was observed after 4-h treatment (Figure 2D). In addition, OSU-03012 inhibited p-AKT (Ser473) starting after 6-h treatment in RH30 cells (Figure 2C).

### OSU-03012 inhibits cell viability in RMS cells

**Figure 3:**
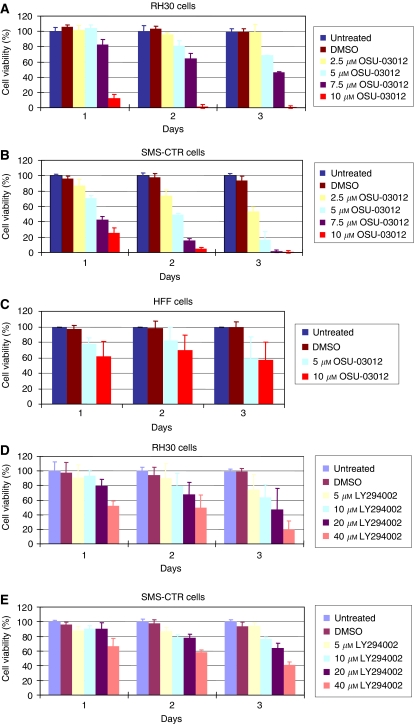
Effects of OSU-03012 and LY294002 on cell viability (**A**–**E**). RH30 (**A**), SMS-CTR (**B**) and human fibroblast (HFF) (**C**) cells were exposed to OSU-03012 at the designated concentrations and cell viability was evaluated on days 1, 2 and 3, respectively. There was a dose-dependent decrease in cell viability upon treatment with increasing concentration of the inhibitor. Human fibroblast is much less sensitive to OSU-03012 compared to rhabdomyosarcoma cell lines. RH30 and SMS-CTR cells were treated with Ly294002 at the indicated concentrations and cell viability was evaluated on days 1, 2 and 3 respectively (**D** and **E**).

Next, we examined the inhibitory effect of OSU-03012 on cell viability in RMS cells. RH30 and SMS-CTR cells were treated with OSU-03012 at 2.5, 5, 7.5 and 10 *μ*M respectively for up to 3 days. On each day, cell viability was determined (Figure 3). OSU-03012 reduced cell viability and induced cell death in both RH30 and SMS-CTR cells in a time and dose-dependent manner (Figure 3A and B). There was more than 70% decrease in cell viability of both RH30 and SMS-CTR with 10 *μ*M OSU-03012 compound treatment for 24 h. The IC_50_ value for RH30 is 6.3±1.1 *μ*M and for SMS-CTR is 5.0±1.6 *μ*M. In comparison, human fibroblast (HFF) was much less sensitive to OSU-03012 in cell viability. There was still nearly 60% cell survival upon 10 *μ*M OSU-03012 treatment for 3 days (Figure 3C).

Phosphoinositide-3 kinase is a major upstream activator of PDK-1. We also compared the potency on reducing cell viability of inhibiting PI3-K using an existing and potent PI3-K inhibitor, LY294002 with inhibiting PDK-1 using OSU-03012 in RH30 and SMS-CTR cells. To compare the effect of LY294002 on cell viability to that of OSU-03012, RH30 and SMS-CTR cells were treated with LY294002 at 5, 10, 20 and 40 *μ*M concentration for up to 3 days. Since PI3-K is an upstream activator of PDK-1, we predicted that inhibition at PI3-K level may be more potent than inhibition at PDK-1 level. However, to our surprise, LY294002 showed much less inhibitory effect on cell viability compared to the same doses used with OSU-03012 (Figure 3D and E). Our results suggested that the IC_50_ of OSU-03012 was four times lower in RH30 cells and nine times lower in SMS-CTR cells than LY294002 (data not shown). These results indicate that novel OSU-03012 compound may be a more potent inhibitor to PDK-1/AKT pathway than the existing PI3-K inhibitor, LY294002 to treat RMS.

### OSU-03012 induces apoptosis in RMS cells

**Figure 4:**
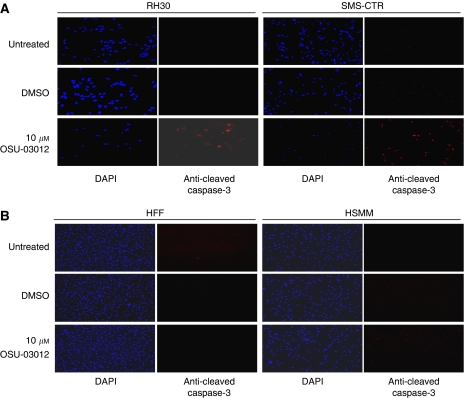
Effects of PDK-1 inhibitor OSU-03012 on apoptosis induction. (**A** and **B**) The cells were treated with 10 *μ*M OSU-03012 for 24 h and fixed. Caspase-3 cleavage (shown in red) was evaluated by immunofluorescence staining with anti-cleaved-caspase-3 antibody. 4′,6-Diamidino-2-phenylindole dihydrochloride (DAPI) staining is shown in blue. Upon OSU-03012 treatment, RH30 and SMS-CTR cells showed an increased amount of caspase-3 cleavage (**A**). However, human fibroblast (HFF) and human skeletal muscle myoblast (HSMM) cells did not show any detectable caspase-3 cleavage after treatment (**B**).

To explore whether OSU-03012 induces apoptosis in RMS cells, we examined levels of cleaved-caspase-3 (a biomarker for apoptosis) by immunofluorescence. OSU-03012 treatment resulted in increased cleaved-caspase-3 in both RH30 and SMS-CTR cells (Figure 4A). However, OSU-03012 did not induce any detectable apoptosis in normal HFFs and normal HSMMs, which are negative controls for RMS cells (Figure 4B). Western blotting showed that OSU-03012 treatment led to cleavage of PARP in both RH30 and SMS-CTR cell lines (Figure 2A and B), which is an other evidence of apoptosis. These suggest that OSU-03012 treatment results in apoptosis in both RMS cell lines RH30 and SMS-CTR.

## DISCUSSION

AKT plays a critical role in controlling the balance between cell survival and apoptosis. The activation of AKT signalling pathway has been reported in several types of cancers. In this study, we explored if the status of AKT level influences cell survival in RMS. Here, we report that AKT phosphorylation is elevated in both RMS tissue samples and cell lines. Interference of AKT signalling pathway with OSU-03012, a small molecule compound targeting PDK-1 activity (Zhu *et al*, 2004), inhibited cell growth and induced apoptosis in RMS cell lines, RH30 and SMS-CTR.

Elevation of AKT phosphorylation at both phospho-sites Ser473 and Thr308 is found in both ARMS and ERMS tissues as well as cell lines. Among 32 ARMS tissue samples, 43% are positive in p-AKT^ser473^ antibody immunostaining; whereas 42% are positive in p-AKT^Thr308^ antibody immunostaining. Among 32 ERMS tissue samples, 55 and 35% are positive in p-AKT^ser473^ and p-AKT^Thr308^ antibody immunostaining respectively. We analysed the association between positive immunostaining (score=2 or 3) with the stage or the subtype of the tumour, but no statistically significant correlation was found. This might be due to the limited patient sample number in our study. However, there is an apparent association between p-AKT^ser473^ and p-AKT^Thr308^ antibody positive immunostaining (*P*<0.05) in both subtypes of RMS. Elevated p-AKT levels are also found in RMS cells. Phosphorylation at both threonine 308 and serine 473 are required for the activation of AKT. Elevation of AKT phosphorylation may be due to any activation of upstream protein kinase receptor Her2 or EGFR which activates AKT via PDK-1 in RMS (Zhou *et al*, 2004). In addition, reduction in activity of the negative regulator of AKT signalling, such as PTEN, might also contribute to the elevation of p-AKT. However, PTEN protein levels of most RMS cell lines we tested are similar as normal cells except that of RH30 and RH3 are at very low level similar to a PTEN-defective MDA-MB-468 breast cancer cell line (data not shown) (deGraffenried *et al*, 2004). Further studies are necessary to elucidate the underlying mechanisms leading to activation of AKT signalling in RMS.

Our data suggest that RMS cell lines that express high level of phospho-AKT, at least RH30 and SMS-CTR, seem to depend on AKT pathway for cell proliferation and survival. As shown here, intervention of AKT signalling pathway using a small molecule compound OSU-03012 targeting PDK-1, an upstream regulator of AKT, inhibited cell survival. OSU-03012 was potent in inhibiting cell growth in both RH30 and SMS-CTR with the concentration resulting in 50% growth inhibition values of 6.3 and 5.0 *μ*M, respectively. OSU-03012 was also able to effectively induce apoptosis in both RH30 and SMS-CTR through caspase-3 and PARP cleavages. Further, we observed that OSU-03012 treatment was able to repress phospho-AKT levels in both RH30 and SMS-CTR cells. In this case, PDK-1/AKT pathway may serve as a potential therapeutic target in RMS cells. Interestingly, OSU-03012 also slightly inhibited the phosphorylation of AKT at both Ser473 and Thr308 sites (data not shown). Since cell viability assay in our study showed that OSU-03012 treatment has little effect on HFF cells, it suggests that PDK-1/AKT pathway may not be essential for the survival of normal cells, such as HFF. In contrast, PDK-1/AKT pathway is indispensable for the survival of cancer cell lines, such as RH30 and SMS-CTR.

Importantly, compared to the effects of LY294002, a potent PI3-K/AKT signalling pathway inhibitor (Altomare *et al*, 2004), IC_50_ of OSU-03012 is several folds lower in RMS cells. These results indicate that novel OSU-03012 compound may be a more potent inhibitor to PDK-1/AKT pathway than the currently potent inhibitor of PI3-K/AKT, LY294002 to treat childhood RMSs. Further, although OSU-03012 is effective in inducing apoptosis in RH30 and SMS-CTR cells (Figure 4A), it did not induce detectable apoptosis in normal human cells such as HSMM and HFF (Figure 4B). Therefore, an orally active compound, OSU-03012 may have a future clinical application for the treatment of both ARMS and ERMS that express activated PDK-1/AKT signalling pathway.

## Figures and Tables

**Table 1:** Clinicopathological parameters of ARMS and ERMS patients used

	**Number of patients**	
**Variable**	**ARMS (*n*=32)**	**ERMS (*n*=32)**
*Gender*		
Male	13	24
Female	19	8
		
*Age (years)*		
0–4	11	18
5–14	14	10
15–19	7	4
Mean	9.1	5.8
Median	10	4
		
*Stage*		
I	5	7
II	5	4
III	15	14
IV	7	7
		
*Primary site*		
Head and neck	9	10
Extremity	13	3
Genitourinary	7	10
Others	3	9

**Table 2:** Elevated phosphorylation levels of AKT in ARMS and EMRS patients^a^

	**Positive (%)**	
	**ARMS (*n*=32)**	**ERMS (*n*=32)**
p-AKT (T308)	42	35
p-AKT (S473)	43	55

a The immunostaining intensity was scored under the microscope on the following scale: 0, no staining; 1, weak staining; 2, moderate staining and 3, intense staining. Scoring of the tissue microarray was completed by at least two independent researchers. Scores of normal tissues are 0 or 1. Scores of 2 and 3 of IHC staining in ARMS and ERMS tissues were considered as positive. % positive: percentage of numbers of positive divided by the total number of specimens.
